# Gender identity is indexed and perceived in speech

**DOI:** 10.1371/journal.pone.0209226

**Published:** 2018-12-20

**Authors:** Melanie Weirich, Adrian P. Simpson

**Affiliations:** Institute for German Linguistics, Friedrich-Schiller University, Jena, Germany; Vita-Salute San Raffaele University, ITALY

## Abstract

This study investigates a possible relationship between perceived and self-ascribed gender identity and the respective acoustic correlates in a group of young heterosexual adult speakers. For the production study, a sample of 37 German speaking subjects (20 males, 17 females) filled out a questionnaire to assess their self-ascribed masculinity/femininity on two scales. A range of acoustic parameters (acoustic vowel space size, fundamental frequency, sibilant spectral characteristics) were measured in speech collected from a picture describing task. Results show that male speakers judging themselves to be less masculine exhibited larger vowel spaces and higher average fundamental frequency.For the perception experiment, a group of 21 listeners (11 males, 10 females) judged masculinity of single word male stimuli drawn from the collected speech sample. A significant correlation between speakers’ self-ascribed and listeners’ attributed gender identity was found with a stronger relationship for female listeners. Acoustic parameters used by listeners to attribute gender identity include those used by speakers to index masculinity/femininity.The investigation demonstrates the importance of including self-ascribed gender identity as a potential source of inter-speaker variation in speech production and perception even in a sample of heterosexual adult speakers.

## Introduction

The traditional gender dichotomy is being increasingly called into question, both in popular discourse as well as in the scientific domain. A popular scientific magazine [1] dedicated a complete issue to the many facets of gender identity and its shifting understanding. Questions of sex and gender have also featured heavily in recent political debate, giving rise in some cases to a demand for a change in the law. For example, in 2017 the German Federal Constitutional Court demanded the need for a tertiary categorization of gender to be made available for the official registration of a person’s gender. Scientific studies also paint a more gender diverse picture than the surface gender dimorphism might at first suggest, since being a woman or a man comprises both gender and sex and reflects sociocultural, learned and biological factors [2,3]. Even when traditional male-female group categorization is adhered to, studies find a great deal of within-group variation in gender identity [4,5] which can be attributed to both behavioral and physiological factors while it is still unclear how the two interact. Indeed, it has been suggested that effects of behavior on hormones are stronger than the effects of hormones on behavior [6,7]. For example, it has been found that caring parenting behavior decreases the testosterone levels in men [8].

What about the impact of behavioral and physiological factors on gender-specific differences in *speech*? To answer this, we first have to look at which differences have been found between the sexes in terms of acoustic-phonetic speech patterns. Many studies have investigated the potential biological reasons for variation in male and female speech (e.g. [9–11]). We know that differences in average male and female fundamental frequency are in part due to average differences in length and mass of the vocal folds [12]. Likewise, the breathier voice quality repeatedly reported in female voices has been suggested to be grounded in differences in the thickness of the vocal folds that affect the closing behavior of the vocal folds with a constant airflow between the thinner vocal folds of females [10,13]. The higher formant values found in females can be attributed in part to overall length differences between male and female vocal tracts, more subtle differences possibly due to the varying dimensions of oral and pharyngeal cavity length [9,14]. Other studies, however, have shown that cross-language differences in the size of inter-gender differences point to an equally important behavioral/learned component. For example, [15] discovered larger inter-gender differences in fundamental frequency (f0) in Japanese speakers than in Dutch speakers. Interestingly, this was associated with culture-specific values ascribed to f0 variation, since for Japanese listeners f0 was more important for attractiveness ratings than for Dutch listeners. [16] shows that the differences between the size of the female and male acoustic vowel space differs from language to language, again implying a learned component.

Regarding German, the language investigated in this study, alongside the expected differences regarding f0 patterns between male and female speakers [17], acoustic-phonetic differences have been found regarding vowel space size [18] and spectral characteristics of sibilants [19]. F0, vowel space and sibilant characteristics were thus considered relevant analysis parameters for the present production and perception study.

People learn by observing and mimicking (e.g., Social Learning Theory [20]), children learn syntactic, prosodic and phonological structures of their mother tongue by imitating the people around them. Besides dialectal pronunciation patterns, children also learn socially relevant fine phonetic detail [21–23]. For instance, [23] found that boys and girls from Newcastle use a gender-specific variant of medial /t/ already from age 3–4. Also, differences in fundamental frequencies and formant values found between preschool boys and girls suggest that anatomical reasons cannot account for gender-specific variation in speech [22,24,25]. That this learning of indexical fine phonetic detail does not end with puberty but persists during adult life was shown convincingly by [26], who examined the tensing of /ɪ/ in Standard Southern British (known as *happ****Y****-tensing)* and could show that even Queen Elizabeth II, one of the subjects in the study, has participated in this process of sound change.

When it comes to gender-specific speech patterns the problem is that most linguistic studies still assume that women and men are homogeneous groups. One exception is the research area focusing on differences within women and men based on the sexual orientation of the speaker (e.g. [27], for an overview see [28]). For example, the frontal articulation of /s/ (lisping) has been found to be associated with stereotypical gay speech, and can be used by listeners to identify a speaker’s sexual orientation but also gender identity in gay and transgender men [29–34]. Gender identity in children (assessed by a parent-filled questionnaire) was found to play a role in the development of gender differences in adolescent boys in /s/ production [35].

Languages can differ in the way they transport sexual orientation [36]. This is suggested by culture-specific stereotypes, such as those just mentioned, as well as by empirical acoustic analyses [36,37]. Of immediate interest are two recent studies dealing with acoustic correlates of sexual orientation and gender identity in German speaking subjects [37,38]. Results suggest that gender identity is an important additional factor besides sexual orientation also in adult speakers. [38] found that while differences between lesbians and straight women were sometimes lacking, within the group of lesbians the first two formants (F1 and F2) in /iː/ and median f0 varied depending on gender-role self-concept. Also, the more feminine gay men described themselves, the higher their F1 and F2 in /uː/, the higher the center of gravity (CoG) in /ʃ s/, the longer the voice onset time (VOT) in /t/, and the aspiration in /k/. A first hint that gender identity also plays a role in straight men was given by the result that the more feminine gay but also straight men described themselves, the higher their mean F2 (of /i a u/) [37].

To summarize, a speaker’s sex, their sexual orientation and gender identity in transgender people, children, lesbians and gay men have been shown to be reflected in speech. However, the potential role of gender identity in adult heterosexual speakers has been largely neglected. Research has shown that there is considerable variation in self-ascribed gender identity within groups of straight women and men [4,5]. The present study goes some way to correcting this imbalance, and, to our knowledge, it is the first larger acoustic study that investigates the relationship between self-ascribed gender identity and fine phonetic detail in the speech of straight men and straight women.

In addtion, a listening test is conducted to reveal if listeners actually *hear* the self-ascribed gender identity of a speaker. In other words, is there a strong relationship between self-rated and perceived gender identity? Studies dealing with perceived gender identity concentrate on the construct of sounding masculine (or macho) [39–43]. Several parameters have been found to be relevant for the perception of more or less masculinity in voices. These comprise mean fundamental frequency, variation in fundamental frequency, vowel space size and the spectral characteristics of /s/ [39–44]. Again, this seems to be language specific, as suggested by the results of studies on various languages. For instance, while spectral characteristics of /s/ have repeatedly been found to be relevant in studies of British or American English [32–33,44] as well as Italian [36], patterns in German, however, are less clear. So, while listeners’ judgments of sexual orientation correlated with spectral characteristics of /s/ in one study [37], this was not the case in another which compared German and Italian [36]. Similarly, studies suggest that vowel formants play a bigger role in English and German than they do in Italian [30,36].

Furthermore, the differences just described appear to be present from an early age. In pre-pubertal American English speaking children–where anatomical differences between the sexes in the speech apparatus are still missing—listeners use the variation in formant frequency spacing to make sex and masculinity/femininity judgments [45]. [46] extended this line of research by investigating not only the perception of gender typicality in children diagnosed with and without gender identity disorder (GID) but also its potential manifestation in production and found a range of acoustic cues (f0, formants and sibilant characteristics) to index gender (a)typicality.

The question arises why gender identity may be indexed in speech at all? The motivation behind expressing gender identity in speech has been studied mostly with respect to transgender individuals or sexual orientation [47,48,49]. [47], for example, found that an important factor for gay men to index their sexual orientation in their speech (and as a result be perceived as gay) is their—positive or negative—attitude towards “sounding gay”. [48] is especially relevant in the context of the present study since they also included heterosexual speakers. In general, men believed their voices were more revealing than women did. The authors considered this to be a possible indicator of more marked stereotypes in connection with gay voices (see also [50]). Interestingly, masculine-sounding *heterosexual* men were the ones with the strongest wish to disclose their sexual orientation. The authors suggest this might be due to the fact that this group has “the most status to defend by signaling their heterosexuality clearly to others” (p.62) (see also [51,52]. A second potential explanation suggested might be that this group of speakers is keen to maximize the probability of finding a female partner. This in turn is supported by a study showing that females interested in mating do indeed use nonverbal cues to detect sexual orientation [53]. Another study takes this a step further by looking at the relationship between self-rated and perceived *gender typicality* in a group of males [49]. Significant correlations were found both between sexual orientation and gender typicality ratings as well as between self-rated and perceived gender typicality ratings.

The present study further investigates the complex relationships between the self-rated gender identity of a speaker, potential acoustic cues and their perception by listeners in a group of heterosexual adults. We test the following hypotheses. First, we predict that the gender identity of heterosexual adult speakers is indexed in their voices. Hence, we explore which acoustic-phonetic cues vary depending on the speakers' self-ascribed gender identity. Second, we predict that listeners are able to use acoustic-phonetic cues to *attribute* gender identity to a target speaker. We investigate which phonetic cues are used by listeners. Third, we predict a positive relationship between self-ascribed and perceived gender identity in male speakers. In addition, we explore whether the listener’s gender plays a role.

In our *production* study we investigate phonetic variation within the same gender. Specifically, do men who rate themselves as very masculine also index this in their speech? And if so, using which parameters? In our *perception* study, we investigate whether this self-rated gender-identity is perceived by listeners. Combining the findings of the two studies, we ask whether the acoustic cues used for indexing and attributing gender-identity are essentially the same?

### Overview

This investigation consists of a production study and a perception study examining the potential acoustic-phonetic cues associated with self-rated and perceived gender identity. The *production* study focusses on finding acoustic parameters that vary within the same gender due to differences in self-ascribed gender identity ratings (male and female subjects). The speech material consists of utterances of words extracted from unscripted speech and the parameters investigated comprise mean fundamental frequency, variation in fundamental frequency, acoustic vowel space size and the acoustic contrast between the sibilants /s/ and /∫/.

The *perception* study concentrates (due to the results of the production study) on male speakers. The speech material used consists of a short bisyllabic word only and its aim is twofold: first, to assess the relationship between self-ascribed and perceived gender identity; second, to examine the acoustic cues potentially responsible for the perceived gender identity ratings. These cues are linked to the production study and comprise mean fundamental frequency, fundamental frequency pattern, first and second formant of /a/, spectral characteristics of /s/ and duration.

### Ethics statement

Written consent from all subjects taking part in the production and perception study was obtained prior to the recording of the stimuli and the perception test. The data collected and experiments conducted were part of a larger project which was reviewed and approved by the Ethics Committee of the University of Jena.

## Production study

### Material and methods

#### Participants

The data used in this study is drawn from a larger project investigating the speech of mothers and fathers to their child and other adults over the timespan of their child’s first year [54,55]. Due to their participation in the project all speakers were living in a heterosexual relationship and expecting their first child. The data presented here were collected approximately four weeks before the expected time of birth. Participants received 160 Euro (approximately 185 US dollars) as compensation for their participation in the whole project including four recording sessions over the course of one year, and all participants were assured that data would be treated confidentially and used solely for scientific purposes.

Participants were asked to fill in a questionnaire regarding their demographics and factors related to the project (biological sex, age, place of birth and residence, nationality, mother tongue, family status, occupation, children, date of expected child, planned parental leave, known speech/hearing disorders). The gender/sex options participants were given included “female”, “male” and “others”. No participant chose the last option. No explicit data regarding sexual orientation were obtained from the subjects. Data from 37 German subjects (20 males and 17 females, mean age 29.3 years (SD 4.9)) were collected. All speakers live in a small city (Jena) in East Central Germany, exhibiting mild dialectal influence.

#### Speech material

**Fig 1:**
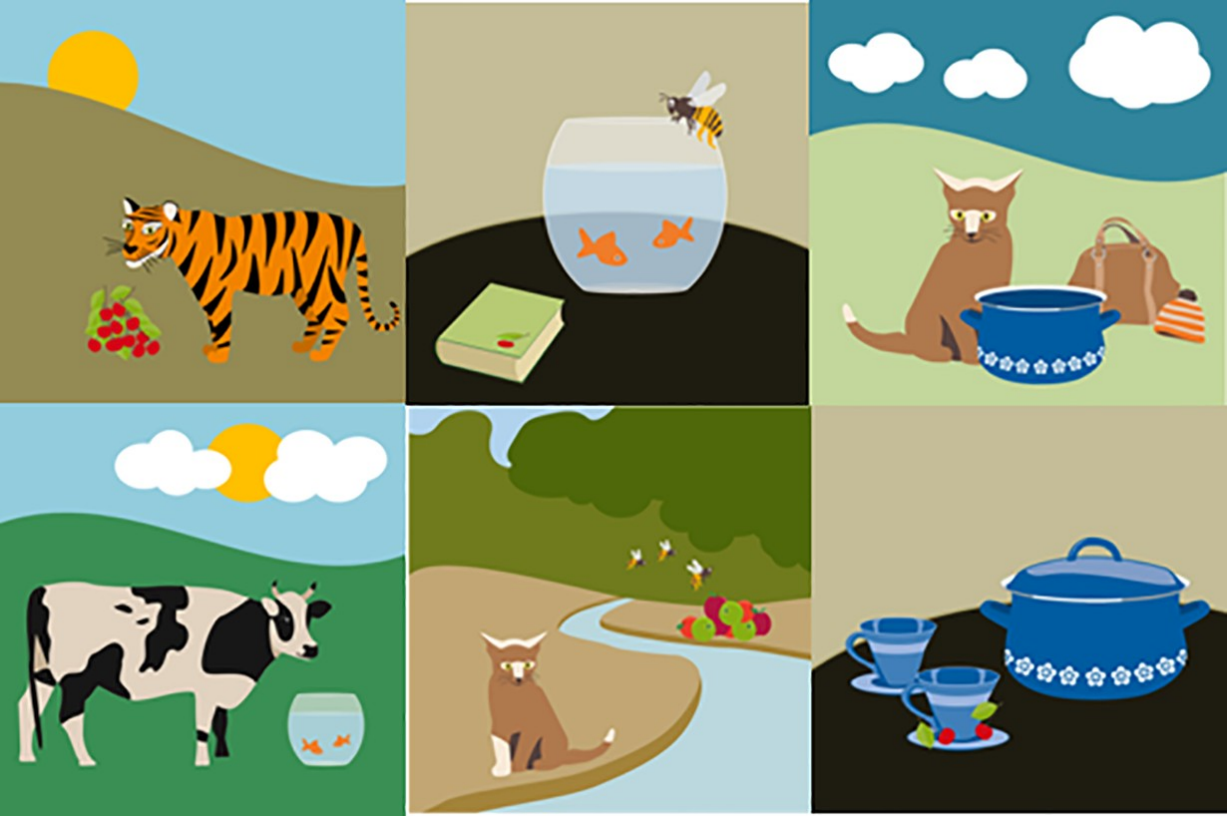
Speech material. A selection of pictures used in the picture describing task to elicit carrier words with embedded target sounds.

Participants were recorded at home by the same female experimenter using a headset-microphone (Sennheiser ew 100 G3 –SK100) and a ZOOM–H6 Handy Recorder. A picture describing task was used to elicit unscripted speech using 15 pictures with various objects and animals (see Fig 1). The experimenter asked the participant to describe the content of each picture. The mean duration of the recordings was 390s (SD: 153s).

The pictures were created to elicit each target word at least three times and comprised the sibilant /s/ in the words *Tasse* (cup) and *Fluss* (river), the sibilant /ʃ/ in the words *Tasche* (bag), *Fisch* (fish) and *Kirsche* (cherry), and the vowels /i: ɛ a u:/ in the carrier words *Tiger* (tiger), *Biene* (bee), *Äpfel* (apples), *Katze* (cat), *Wasser* (water), *Kuh* (cow), *and Buch* (book). Table 1 gives an overview of the target words and sounds and the number of tokens included separated by gender and sound.

**Table 1:** Overview and number of carrier words and target sounds.

Target sound	iː	ɛ	a	uː	s	ʃ
Carrier word	*Tiger*	*Äpfel*	*Katze*	*Kuh*	*Tasse*	*Tasche*
	*Biene*		*Tasse*	*Kuchen*	*Fluss*	*Fisch*
	*Wiese*		*Wasser*	*Buch*		*Kirschen*
Number of tokens (f/m)	180/254	74/95	137/202	183/227	182/214	351/517

#### Gender identity

Self-reported gender identity ratings were obtained using two different questionnaires. The F+ scale of the German version (GEPAQ-F+, [56]) of the Personal Attributes Questionnaire [57] consists of 7 item pairs concerning positive attributes traditionally associated with females, e.g. aware/not aware of feelings of others, very/not at all kind, very gentle—very rough. Each item pair is rated on a scale from 1–7 with high numbers reflecting high self-ascribed feminine attributes.

The second questionnaire used is the Traditional Masculinity-Femininity scale (TMF), an instrument recently developed for measuring gender-role self-concept [5]. The TMF claims to assess the constructs of *gender-role adoption (*actual manifestation of how masculine-feminine a person considers her- or himself to be), *gender-role preference* (desired degree of masculinity-femininity), and *gender-role identity* (comparison of gender-related social norms and the gender-related characteristics of the individual). Participants have to index for 7 items on a scale from 1 (very masculine) to 7 (very feminine), e.g. how their interests/behaviour/appearance would be evaluated tradionally, how they consider themselves, and how they would ideally like to be. In other words, the higher the scores, the higher the speaker’s self-ascribed femininity.

Reliability of the two scales was measured by calculating Cronbach’s α using the *psych* package in R. For both scales reliability was high, with higher values for TMF than for GEPAQ-F+ (Cronbach’s α = .94 and = .80).

Both questionnaires were filled in by the participants after the recordings. For each scale (GEPAQ and TMF) a mean score was calculated for each speaker and used for the correlations with the listeners’ ratings and the acoustic measures.

#### Acoustic analyses

Acoustic segmentation and analysis was done using PRAAT (version 6.0.19, [58]). Start and end of carrier words and target sounds were labeled. For both vowels and sibilants, labeling was oriented on the envelope of the oscillogram. For vowels, the formant structure, for sibilants the energy distribution visible in the spectrogram was also taken into account. Acoustic parameters were analyzed that have repeatedly been shown in other studies to indexicalize different aspects of group identity, such as gender and sexual orientation (see above).

The first three formants of /i: ɛ a u:/ were measured in the middle of the vowels using PRAATs LPC formant measurement algorithm (analysis parameters: time step = 0.01s, maximum number of formants = 5, window length = 0.025s, preemphasis from = 50 Hz) The maximum formant value was set to 5000 Hz for the male speakers and to 5500 Hz for the female speakers. Formants were manually corrected if necessary. Mean F1 and F2 values of each vowel category for each speaker were used to calculate the size of a speaker’s acoustic vowel space. This vowel space was estimated by calculating the area of the polygon enclosed by the mean formant values of the vowels in the F2xF1 space. We used Bark, a psycho-acoustic measure better reflecting the non-linearity of the perceptual system [59].

Discrete Cosine Transformation (DCT, [60]) was used to parameterize the spectra of /s/ and /ʃ/ using the EMU package of the R software [61]. DCT has been shown to be a useful technique to effectively separate the four fricative types in Polish [62], and to be a reliable parameter to differentiate the very similar acoustic spectra of /ç/ and /ʃ/ in different varieties of German [63]. In this case, the DCT decomposes the spectrum into a set of half-cycle cosine waves and the resulting amplitudes of these cosine waves are the DCT coefficients (corresponding to the cepstral coefficients of a spectrum). Three DCT coefficients were used for the analysis: DCT1 is proportional to the linear slope of the spectrum, DCT2 corresponds to its curvature and DCT3 describes the amplitude of the higher frequencies. Mean values for each speaker and sibilant were calculated for the three coefficients and Euclidean distances (EDs) in the mel-scaled DCT1xDCT2xDCT3 space were calculated for each speaker to quantify the acoustic contrast between /s/ and /ʃ/. Mel-scaling of the spectra was chosen since this has been found to optimally describe the acoustic distinction between sibilants (e.g. [64]).

Furthermore, mean fundamental frequency (f0) and standard deviation of f0 were measured over the whole utterance of the speaker (in PRAAT, analysis parameters: time step = 0.01, minimum pitch = 75 Hz, maximum pitch = 600 Hz).

#### Statistical analysis

Welch Two Sample t-tests were run to investigate gender differences in the self-rated GEPAQ and TMF scores of the speakers. To analyze the relationship between the two scores and between acoustic cues and self-rated gender identity scores, Pearson Product Moment correlations (one-sided) were run. Since correlation analyses were run for several acoustic parameters and both genders separately, p-values were corrected for running multiple tests.

### Results: Indexing gender identity

#### Self-rated gender identity

**Fig 2:**
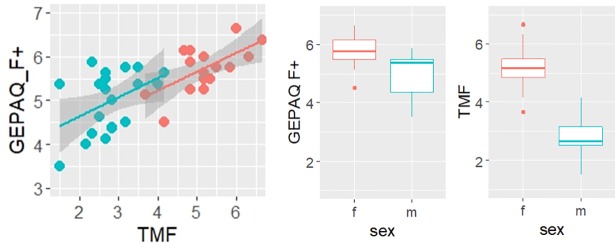
Self-rated gender identity. Left plot: Relationship between GEPAQ_F+ and TMF scores of 20 male (blue) and 17 female (red) speakers. Right plots: Distribution of GEPAQ_F+ and TMF scores: the horizontal line marks the median of the group (females: red, males: blue), the boxes comprise 50% of the data: The whiskers extend to the most extreme data point, which is no more than 1.5 times the interquartile range from the box; outliers are marked with dots.

Fig 2 shows the gender identity scores of the male (blue) and female (red) participants. TMF and GEPAQ significantly correlate for both genders: r = .61, p < .01 (for females), r = .45, p < .05 (for males) (cf. Fig 2 left plot). The relationship is less strong in males, which is also mirrored by the large variation in the GEPAQ scores with similar TMF scores, e.g., around the mean value of 2.8. Both scores differ significantly between the genders (Welch Two Sample t-tests, p < .001) but the mean values are further apart for the TMF scale (m: 2.8 vs. f: 5.2) than for the GEPAQ scale (m: 5.0 vs. f: 5.7) (cf. Fig 2 right plots). Thus, the TMF scale seems to be a very appropriate scale to differentiate heterosexual German men and women, while there is more overlap between the genders in the GEPAQ scale.

Although TMF does a better job in distinguishing the genders, GEPAQ scores are more widely distributed *within* the genders (and particularly in the male group), thus providing a better starting point for correlations with the acoustic parameters. For this reason, unless specified otherwise, subsequent analyses will be carried out using the GEPAQ scores as a measure of gender identity.

#### Acoustic cues of gender identity

**Fig 3:**
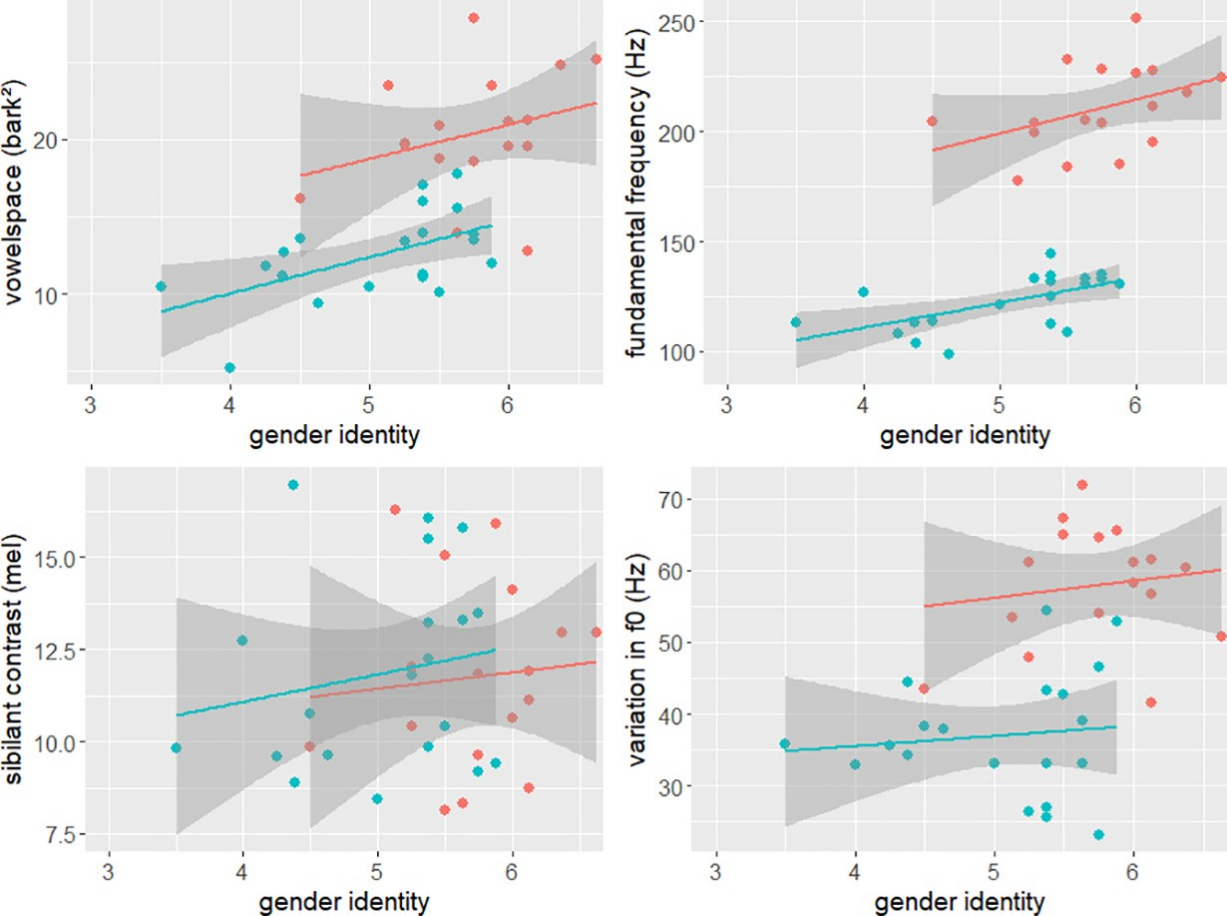
Relationship between self-rated gender identity and acoustic cues. Vowel space size, mean fundamental frequency, sibilant contrast and variation in f0 as a function of self-rated gender identity (GEPAQ_F+) separated by gender (red = females, blue = males). High ratings on the x-axis signify higher femininity ratings.

The investigated acoustic cues comprise the vowel space size, the mean fundamental frequency and the variation in fundamental frequency, and the sibilant contrast. Fig 3 shows the distribution of these acoustic measurements for all speakers (with male speakers in blue and female speaker in red) as a function of the gender identity of the speaker (estimated by means of their GEPAQ score). It can be seen that differences between the genders exist in vowel space size and both fundamental frequency measures but not in the realization of the sibilant contrast. Also, patterns differ regarding the relationship between the acoustic cues and the gender identity scores within a gender. Positive relationships can be seen for the vowel space size and the mean fundamental frequency. For sibilant contrast and variation in f0 the relationships seem less clear.

Pearson's product-moment correlations were run for each acoustic parameter and both genders and Table 2 gives a summary of the results. As was already clear from the figures, no significant correlation was found for the sibilant contrast for either of the two genders. Also, the gender identity did not have a significant effect on the variation in f0. For vowel space size and mean fundamental frequency, however, significant correlations were found for male speakers, with r = .54 and r = .60, respectively, while no effect was found for females.

**Table 2:** Relationship between gender identity and acoustic cues.

*Gender*	*Relationship between*	*r*	*p-value*	*p-value (corrected)*
*males*	GEPAQ & vowel space	.54	0.0061	< .05 *
*females*	GEPAQ & vowel space	.29	0.1254	n.s.
***males***	**GEPAQ & mean f0**	**.60**	**0.0026**	< .05 *
*females*	GEPAQ & mean f0	.41	0.0515	n.s.
*males*	GEPAQ & SD f0	.11	0.3200	n.s.
*females*	GEPAQ & SD f0	.15	0.2875	n.s.
*males*	GEPAQ & sibilant contrast	.19	0.2131	n.s.
*females*	GEPAQ & sibilant contrast	.09	0.3637	n.s.

Similar results were found with respect to TMF. Here too, significant correlations were found for vowel space and mean f0 in males only. For vowel space size, height and significance level are comparable to those reported for GEPAQ (r = 0.56, uncorrected p-value = 0.0055), while for mean f0 the correlation is weaker (r = .40, uncorrected p-value = 0.038).

## Perception study

### Material and methods

#### Speakers

Due to the results of the production study only male speakers were included in the perception experiment. The speakers consisted of a subset of the male speakers described above (16 male speakers) and an additional data set of male speakers recorded only for the purpose of the perception test (3 male speakers, age range 19–21 years, matched in educational background, also living in Jena). The additional speakers also signed an informed consent form. It should be said, however, that we did not inquire further into the status of the relationships of the additional speakers, since this was not deemed relevant to the investigation at hand (i.e. attribution of masculinity due to acoustic cues).

The reason for not taking all male subjects of the production study were conditions placed on the comparability of the stimuli to be used (see below).

#### Stimuli

In contrast to the production experiment, where the speech material investigated consisted of a stretch of running speech, here only one particular word was used. This was done since we did not want other acoustic cues than the ones we are investigating (at least not more than necessary to represent a natural utterance) to be responsible for the ratings. Therefore stimuli were restricted to the bi-syllabic word *Tasse* (*cup*), carrying an open vowel and a sibilant, taken from the speakers’ recording of the picture describing task (see above). From each speaker one *Tasse* token was chosen. Stimuli were normalized by setting the ampliude to the same level using PRAAT. However, no modification of other prosodic parameters (f0, timing) was undertaken. The selected tokens were uttered in the middle of an utterance and followed by a break. In this way, the occurrence of creaky voice and coarticulatory influences were reduced to a minimum. These conditions on comparability meant that 4 of the original male speakers were excluded from the perceptual study.

The start of *Tasse* was set to the release of the closure of the stop. Each stimulus consisted of two *Tasse* tokens of different speakers with a pause of 0.4s between them. After each stimulus a silence of 0.9s was added followed by a beep (with a duration of 0.5s and a frequency of 880Hz) to signal the start of the next stimulus pair to be rated.

#### Listening experiment

The methodology of the perception test was oriented on an earlier study dealing with perceived tempo [65]. The ‘ExperimentMFC’ (Multiple Forced Choice) facility in PRAAT was used to present the stimuli via loudspeakers to the listeners. All possible pair-wise speaker combinations were created out of the 19 speaker stimuli, excluding same-speaker combinations. This resulted in 171 different pairs. 21 listeners (11 males, 10 females, mean age 22.8 years, SD = 2.3) rated all speaker pairs once in a randomized order. Listeners had to decide which of the two speakers (speaker A or speaker B) sounded more masculine on a scale from 0 to 3. Each stimulus pair was co-presented with the question “Which of the two speakers is more masculine?” on top of the screen and the possible answer buttons to choose of (A3 to B3). Listeners were allowed to choose 0, meaning that they did not judge one of the two speakers to be more masculine than the other, while 3 would indicate that one speaker was judged to be much more masculine than the other. We chose to ask listeners to compare two stimuli rather than individually judge each stimulus on a predefined scale in an attempt to prevent a continuous recalibration of the reference points being used throughout the experiment but also between individual listeners.

Prior to the experiment a test round was carried out with five stimulus pairs to familiarize the listeners with the design. The speakers used for the test round were also part of the main experiment but different *Tasse* tokens were chosen. After a block of 30 pairs listeners could take a break as long as they wanted. Altogether the experiment lasted approximately 30 minutes. After the perception test, listeners were asked to fill in the TMF and GEPAQ questionnaires to examine a possible relationship between listeners’ gender identity and their ratings. Again, reliability of the two scales was measured by calculating Cronbach’s α using the *psych* package in R. Reliability scores were similar to the ones measured for the speakers’ ratings (Cronbach’s α for TMF = .89 and for GEPAQ = .79).

#### Acoustic analysis of stimuli

Parallel to the production experiment, acoustic parameters were analyzed in the *Tasse* stimuli regarding fundamental frequency, formants and spectral characteristics of /s/. In particular, the fundamental frequency in /a/, the difference in fundamental frequency between /a/ and /ə/ (as a coarse measure of the f0 contour of the utterance), the first and second formant of /a/, DCT 1–3 of /s/, and the duration of the word and of /s/ (in ms and in % of the word) were measured.

#### Statistical analyses

Linear Mixed Models as implemented in the *lme4* package [66] in R (version 3.4.2, [67]) were run with the perceived masculinity ratings as dependent variable. As fixed factors we entered the self-rated gender identity scores, listeners’ gender, listeners’ gender identity and the investigated acoustic cues. The listener and speaker 1 and speaker 2 of the rated stimulus pairs were entered as random effects. P-values were obtained using likelihood ratio tests comparing the model with and without the factor (or interaction) in question.

### Results: Attributing gender identity

#### Perceived gender identity

**Fig 4:**
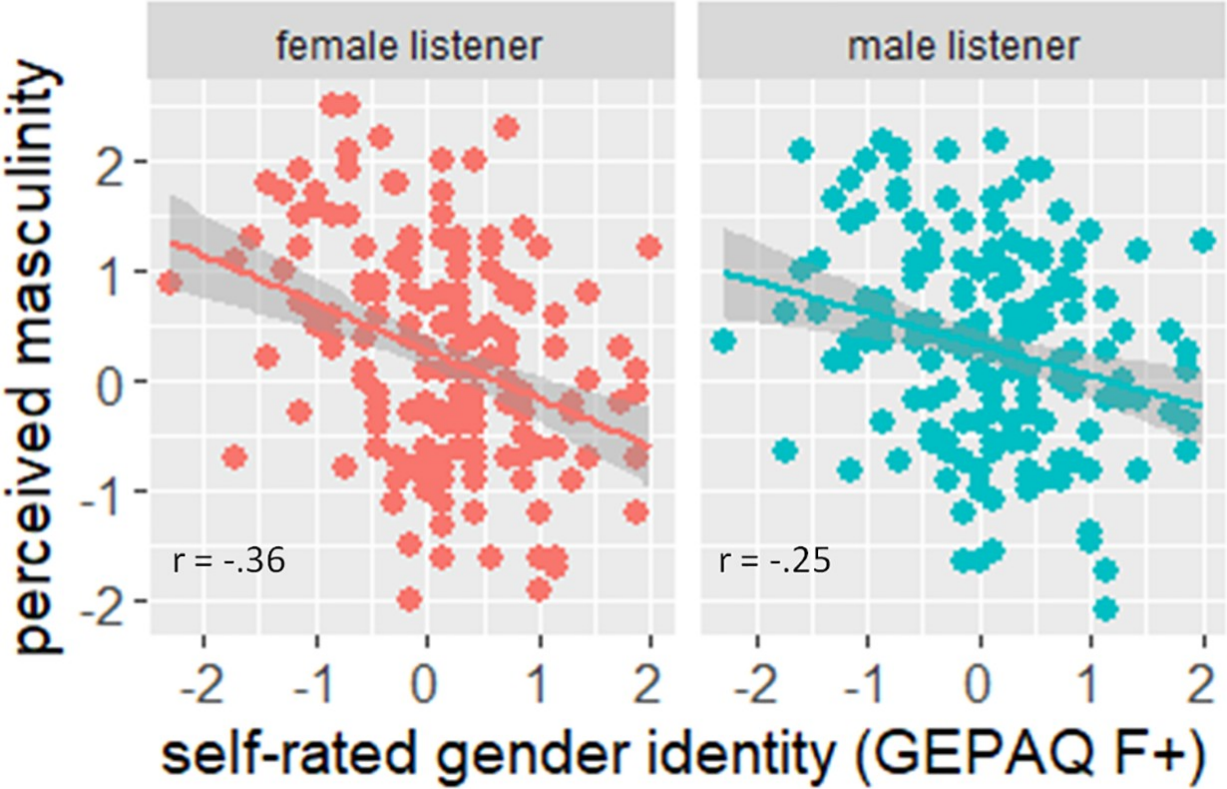
Relationship between self-rated and perceived gender identity. High positive values on the y-axis imply the second speaker to be perceived as more masculine. High positive ratings on the x-axis imply the second speaker to have higher femininity score (GEPAQ_F+). Data distribution and correlation coefficients (Pearson) are shown separated by gender.

A linear mixed model (LMM) with the rated masculinity scores as dependent variable, the GEPAQ scores and the listener’s gender as fixed factors and the listener and speaker 1 and speaker 2 of the stimulus pairs as random effects revealed a significant interaction of the listeners’ gender and the GEPAQ scores of the speaker (χ^2^(3) = 13.82, p < .01). Thus, a relationship between self-rated and perceived masculinity exists and this relationship is affected by the gender of the listener. The data were therefore separated by listeners’ gender and mean scores for each stimulus pair were calculated for the genders, respectively. Fig 4 shows the relationship between perceived (y-axis) and attributed (x-axis) masculinity separately for male (left plot) and female (right plot) listeners. A high positive score on the y-axis means that the second speaker of the respective pair was rated on average more masculine than the first speaker. A high positive score on the x-axis means that the second speaker had higher GEPAQ-scores (higher femininity) than the first speaker. Pearson correlations between listeners masculinity ratings and self-rated gender identity scores were significant for both genders (females: p < .001, males p < .01) but differ in size: female listeners seem to be more sensitive in rating gender identity of male speakers than males since there is a higher correlation (r = -.36 for females vs. r = -.25 for males).

We also checked for an effect of the gender identity score (GEPAQ) of the *raters* on their evaluations, analyzing the possible hypothesis that listeners with higher femininity scores are”better” in rating gender identity independent of their gender. We therefore divided the data in raters with higher femininity scores (> 5.5, also comprising three male subjects) and lower femininity scores (< 5.5 also comprising two female subjects). However, these groups did not differ in the size of the correlations between perceived and self-rated gender identity of the speakers (p < .001, r = -.30 for more feminine raters vs. r = -.31 for more masculine raters).

In addition, we performed a LMM including the raters’ gender identity instead of their gender. Neither a main effect nor an interaction with the speakers GEPAQ score reached significance.

Thus, in the case of male and female listeners rating the masculinity of male speakers, we assume the listeners’ sex and not gender identity to be the contributing factor to the differences in perceptual ratings.

#### Acoustic cues of perceived gender identity

**Fig 5:**
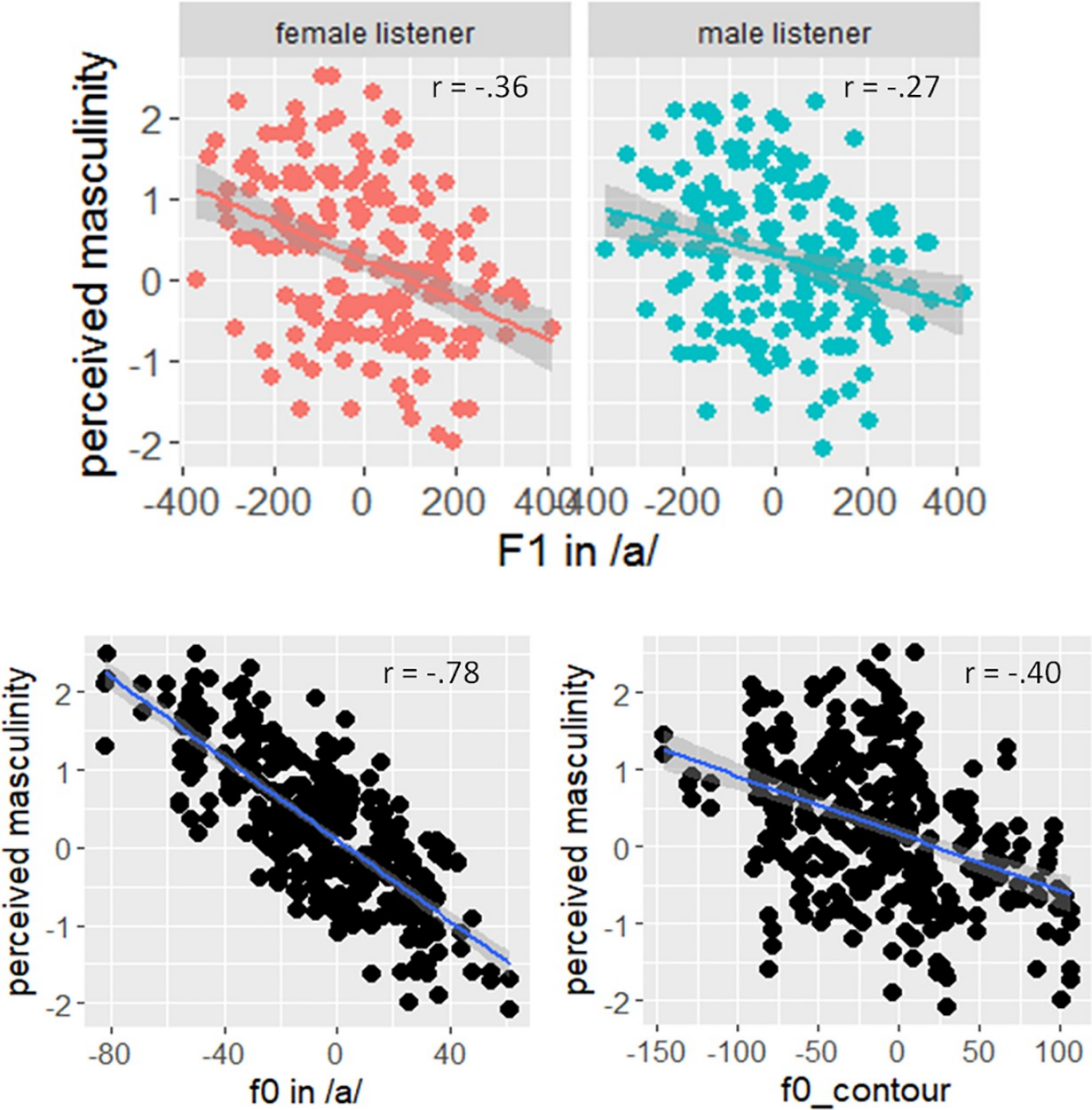
Relationship between perceived masculinity and acoustic cues. Above: Masculinity ratings as a function of F1 in /a/ separated by listener gender (red = female, blue = male). Below: Masculinity ratings as a function of f0 in /a/ (left plot) and f0 contour (right plot). High positive ratings on the y-axis signify that the second speaker of the stimulus pair was rated more masculine, high positive ratings on the x-axis imply that the second speaker had a higher F1, a higher f0 and a rising f0 contour. Pearson correlations coefficients are shown for each relationship.

Several acoustic parameters (and their interaction with listener gender) were entered as fixed factors to the LMM (in a stepwise fashion) to look for a potential impact of this acoustic cue on the perceived masculinity of the speaker. These parameters were the fundamental frequency in /a/, the difference in fundamental frequency between /a/ and /ə/ as a simple quantification of f0 movement over the two syllables, the first and second formant of /a/, DCT 1–3 of /s/, and the duration of the word and of /s/ (in ms and in % of the word duration). Since in the perception test *pairs* of speakers were rated, each investigated acoustic parameter is actually the difference in this parameter between the two speakers. Significant main effects were found for fundamental frequency of /a/ (χ^2^ (1) = 36.55, p < .001) and f0 contour (χ^2^ (1) = 24.2, p < .001). In addition, a significant interaction between F1 and listener gender was also found (χ^2^(2) = 15.25, p < .001). Durational patterns, F2 and spectral characteristics of /s/ did not show a significant effect. Fig 5 visualizes the effects of F1 (above), fundamental frequency (below, left plot) and f0 contour (below, right plot) on perceived masculinity: each dot represents the mean rating of a stimulus pair. A high positive rating on the y-axis implies that the second speaker was rated more masculine, a high positive rating on the x-axis signifies that the second speaker has a higher F1 in /a/, a higher f0 in /a/ and a rising contour. All correlations were significant (p < .001) and negative, meaning that a speaker who was rated as more masculine had a lower F1 in /a/, a lower f0 in /a/ and a falling f0 contour. The interaction between F1 and listener gender is reflected in the fact that the female listeners show a higher correlation than the male listeners, pointing to a stronger influence of F1 on their masculinity ratings. The strongest correlation was found for mean f0 with r = -.78, followed by the f0 contour with r = -.40. Note, that these parameters are independent of each other and not correlated (r = .06, p = .28).

## Discussion

This study highlights three aspects of gender and speech. First, self-ascribed gender identity is not a bimodal factor but a continuous variable which exhibits considerable inter-gender variation. This was most apparent in men’s responses to the GEPAQ questionnaire on positive feminine attributes. Second–and confirming, in part, hypothesis 1 –this idiosyncratic gender identity is indexed in speech through fine phonetic detail: men who rate themselves as more feminine have larger acoustic vowel spaces and higher mean fundamental frequencies (and thereby shifted values in the direction of more typical female values). Third–in support of hypothesis 2 –the concept of masculinity/femininity is attributed by listeners using similar acoustic parameters as those used by speakers (mean fundamental frequency and F1 of /a/), but they also attend to additional parameters (here a rising/falling f0 pattern), probably based on stereotypical assumptions about gendered speech, with female speech being associated with rising f0 contours and male speech with falling contours [68]. Indeed, final f0 rises in questions have been found to be perceived as more hearer-oriented and polite [69] and used more frequently by female speakers [70,71]; see however, [72].

The association of certain spectral cues in speech and perceived masculinity has been found before [39–43,73–75]. Lower fundamental frequency and lower formant values resulting in smaller vowel spaces have been found to correlate with perceived masculinity and thereby increased attractiveness in male voices (e.g. [43,75]). As stated before, differences between languages exist regarding the salience of a particular acoustic cue (e.g. [36]). While characteristics of sibilants seem to be important to index sexual orientation (as found for American English (e.g. [33]) and in some cases also for German [37], but see [36], in the present study, sibilant characteristics in the speech of heterosexual adults were neither found to index gender identity nor used as a cue by listeners to rate masculinity.

Our study highlights an additional gender-specific aspect regarding the transportation of gender identity in speech (in conjunction with hypothesis 3). We found an interaction of formant frequencies and the listener’s gender. Particularly, the acoustic cue of a lower F1 in /a/ (more typically found in male speakers) was used by listeners to attribute masculinity to a greater extent when listeners were female. Together with the finding that females were better raters in ascribing masculinity to male speakers this adds to research dealing with voice characteristics indicating underlying mate quality in humans [74, 76]. It is important to note that gender was the important factor here, not gender identity. This result also adds to the findings of [53] who found that mating interests of women affect their accuracy in judging sexual orientation in male subjects from their faces. In our study we were able to show that not only visual but also auditory aspects are used by females to judge masculinity. In future listening experiments it would be interesting to assess the role of heterosexual females’ mating interests on using acoustic cues to detect sexual orientation and masculinity in males. This, in turn, would build on the finding that female listeners in the fertile phase of their ovulatory cycle are most sensitive to f0 differences in male speakers [77].

While examining the *perception* of masculinity/femininity in speech has a long tradition in linguistic studies, investigating the manifestation of self-ascribed gender identity in speech, and here, particularly in the speech of heterosexual adults, is rather new. Questions of sex and gender have concentrated primarily on the distinction between male and female speakers or have dealt with the sexual orientation of the speakers (gay speakers, lesbians, bisexual speakers or children diagnosed with gender identity disorder, e.g. [30, 33–36, 46]). A more differentiated approach to sexual orientation and gender identity has however been taken in [37,38]. The present study set out to focus particularly on heterosexual adult speakers and showed that also in this—at first sight homogeneous—speaker group, gender identity varies. Moreover, it is not only *attributed* by listeners and thus integrated in the speech perception process, but it is also part of the speech production process being used by male speakers to *index* their self-ascribed masculinity.

We are aware that the production study and the perception study are measuring different facets of the complex construct of gender identity. Self-rated and perceived gender identity were measured differently. Speakers were asked to rate their gender identity on a number of scaled items, whereas listeners were required to compare the masculinity of two stimuli. Even the results from the GEPAQ-F+ and TMF questionnaires illustrate clearly that strongly correlated yet different socio-psychological aspects of gender identity are being assessed (femininity in terms of positive stereotypes and masculinity/femininity in terms of traditional gender roles, see also [5]). The difference between these scales reflects the ongoing debate about the relationship between masculinity and femininity (being either two poles of one dimension or independent constructs, see also [78]. Despite this conceptual complexity it is all the more surprising that speakers’ and listeners’ ratings converge to such a significant extent, at least for male speakers.

Moreover, differences in the correlation between GEPAQ and TMF also exhibited gender-specific patterns with a weaker correlation for men than women. A man scoring low on TMF, and thereby rating himself as very masculine, could still have a high self-rating on the GEPAQ items, such as emotional, helpful, kind and aware of another person’s feelings. This emphasizes the difference between these scales regarding what aspects of gender identity they capture, and this is especially interesting in the light of changing gender roles with men participating more in family life and being more involved in child care than they were a few decades ago. Gender specificity with respect to the importance of gender identity being transported in speech is also reflected in the different correlations between acoustic cues and self-rated gender identity, which were only found to be significant in males.

Both differences in the correlation between GEPAQ and TMF and in the correlations between self-rated gender identity and acoustic cues can be taken as an indication of the importance for males to index their sexuality, gender typicality, masculinity/femininity [48, 50–52, 77].

The perception test was only carried out using male stimuli. This was due to the finding in the production study that only male speakers were overtly indexing their self-ascribed gender identity in their speech. However, future experiments need to test female and male listeners’ judgments of masculinity/femininity in female voices.

Factors such as gender, ethnicity, age, and group membership have played a pivotal role in explaining inter-speaker variability in speech and idiosyncratic fine phonetic detail since the beginnings of sociolinguistics [79]. This study adds to this line of research by highlighting the importance of self-ascribed gender identity to explaining variation in speech production and perception within heterosexual adult speakers.

## Supporting information

S1 Data Gender identity and acoustic measures. (CSV)

S2 Data Perception experiment. (CSV)
